# Early vitrectomy and intravitreal antibiotics for post-operative exogenous endophthalmitis (EVIAN): a randomised control trial of feasibility

**DOI:** 10.1038/s43856-026-01664-w

**Published:** 2026-07-16

**Authors:** Mahiul MK Muqit, Carlos Pavesio, Yanzhong Wang, Stephen Cobb, Christopher Spink, Elena Pizzo, Gerry Clare, Gordon Hay, Hatem A. Wafa, Jiunn Wang, Hayley Boston, Keerththika Sriharan, Early Vitrectomy, Intravitreal Antibiotics for Endophthalmitis (EVIAN) Study Group, David Steel, Tariq Aslam, Irene Stratton, Angie Fletcher, Sir Stephen Cobb, Christopher Spink, Felipe Dhawahir-Scala, Jonathan Smith, Richard Haynes, Teresa Sandinha, Yvonne Luo, Sonali Tarafdar, Kam Balaggan, Mark Costen, Benjamin Clarke, Tim Jackson, Harry Bennett, Bhaskar Gupta, Roxane Hillier, Daniela Vaideanu-Collins, Ahmed Saad, Stephen Winder, Sher Aslam, Mandeep Bhindra, Guy Negretti, Philip Banerjee, Bataung Mokete, James Bainbridge

**Affiliations:** 1 Moorfields Eye Hospital NHS Foundation Trust, City Road, London, EC1V2PD United Kingdom; 2 Institute of Ophthalmology, University College London, 11-43 Bath Street, London, EC1V 9EL United Kingdom; 3 School of Life Course and Populational Sciences, Kings College London, 4th Floor, Addison House, Guy’s Campus, London, SE11UL United Kingdom; 4 The Royal Courts of Justice, Strand, London, WC2A 2LL United Kingdom; 5 Department of Primary Care and Population Health Research, University College London, 1-19 Torrington Place, London, WC1E 7HB United Kingdom; 6 University of Newcastle, Newcastle, United Kingdom; 7 Manchester Royal Eye Hospital, Manchester, United Kingdom; 8 University of Southampton, Southampton, United Kingdom; 9 Health Education England, London, United Kingdom; 10 London, United Kingdom; 11 Sunderland Eye Infirmary, Sunderland, United Kingdom; 12 Bristol Eye Hospital, Bristol, United Kingdom; 13 Royal Liverpool University Hospital, Liverpool, United Kingdom; 14 East Kent Hospitals University NHS Foundation Trust, Canterbury, United Kingdom; 15 Ninewells Hospital, Dundee, United Kingdom; 16 Wolverhampton Hospitals, Wolverhampton, United Kingdom; 17 Hull University Teaching Hospitals NHS, Hull, United Kingdom; 18 Gloucestershire Hospitals NHS, Gloucester, United Kingdom; 19 Kings College Hospital, London, United Kingdom; 20 Princess Alexandra Eye Pavilion, Edinburgh, United Kingdom; 21 Southampton Hospitals, Southampton, United Kingdom; 22 Newcastle upon Tyne Hospitals NHS, Newcastle, United Kingdom; 23 South Tees Hospitals NHS, Middlesbrough, United Kingdom; 24 Sheffield Teaching Hospitals NHS Trust, Sheffield, United Kingdom; 25 Oxford Eye Hospital NHS, Oxford, United Kingdom; 26 Buckinghamshire Healthcare NHS Trust, Aylesbury, United Kingdom; 27 Surrey and Sussex NHS Trust, Redhill, United Kingdom; 28 Frimley Park NHS, Frimley, United Kingdom; 29 Leeds Teaching Hospitals NHS, Leeds, United Kingdom

**Keywords:** Therapeutics, Medical research

## Abstract

**Background:**

Endophthalmitis is a blinding complication following any invasive ocular procedure, and historical treatment guidelines do exist but only for cataract surgery cases. To the best of our knowledge, no randomised controlled trial evidence exists to answer the question of early vitrectomy surgery for the treatment of endophthalmitis following any eye surgery.

**Methods:**

We conducted a multicentre, national prospective randomized controlled clinical (RCT) trial aimed to assess the feasibility of randomizing patients to an early vitrectomy (Arm-A) intervention within 48-96 hours compared to current standard patient care of repeated, immediate intravitreal antibiotic injection (Arm-B) and delayed vitrectomy. There was no blinding or masking conducted in the trial. The key inclusion criteria were: over 18 years of age; confirmed diagnosis of POE at any time-point following an ocular surgery, procedure, or injection; symptomatic visual loss attributable to POE; best corrected visual acuity (VA) worse than 35 ETDRS letters, including counting fingers, hand motions and perception of light vision. The key exclusion criteria were: known adverse reaction to intravitreal antibiotics (amikacin/vancomyin/cephalosporins); optic atrophy in study eye; corneal oedema or haze that would prevent visualisation of fundus to perform vitrectomy surgery. The primary outcome measure was, the feasibility and acceptability of carrying out a RCT of early surgical treatment compared to standard treatment for POE. ClinicalTrials.gov NCT04522661 (13/08/2020).

**Results:**

Here we show that the study is feasible and meets the primary endpoint whereby 63 participants are randomized at 79% recruitment rate. Median change in VA (IQR) as per-protocol from baseline-to-week 24 was 40 (28-70) letters in Arm-A (n = 24) versus 13 (0-66) letters in Arm-B (n = 25); corresponding to a between-group difference of 24 letters, adjusted for baseline, in favour of early vitrectomy (p = 0.139). Higher rate of non-serious adverse events observed in Arm-B (68%) compared to Arm-A (47%), with higher incidence of retinal detachment 8 cases (24%) in Arm-B versus 2 cases in Arm-A (6.7%).

**Conclusions:**

The EVIAN trial meets the primary endpoint demonstrating feasibility in conducting a trial to randomize the target population at multiple eye centres. Although not statistically significant, the effect on VA change indicates a potential meaningful benefit of vitrectomy compared to standard of care treatment, which can now be tested in a definitive phase 3 clinical trial.

## Plain language summary

This study is testing the feasibility of a larger trial of prompt eye surgery for a rare complication of eye surgeries known as “endophthalmitis”, which can occur due to bacterial infection. Currently, people with endophthalmitis receive an antibiotic injection, however this is often not sufficient to control the infection. The physical and psychological impact on the well-being of patients and their carers is immense. A type of surgery known as “vitrectomy” can help relieve this complication if antibiotics do not successfully treat an infection, however it is unknown whether patients would benefit from vitrectomy early, when it is unclear whether or not antibiotics will resolve the infection. We conducted a clinical trial, randomly allocating participants to either have vitrectomy performed as soon as endophthalmitis was diagnosed, or after antibiotics were found not to work. The study was able to demonstrate the feasibility of a larger trial, and indicated early promise for the technique. We are now planning a larger trial to assess whether early surgery is beneficial as a treatment for people with endophthalmitis.

## Abstract (web summary)

Muqit et al. perform a randomized control feasibility trial early vitrectomy of patients with endophthalmitis following eye procedures. They recruit patients successfully, and show a signal of effect from early vitrectomy on visual outcomes with an acceptable safety profile.

## Introduction

Endophthalmitis is a rare but potentially blinding eye complication that may occur following any invasive ocular procedure. Invasive ocular procedures are now very common, more so than ever before. Cataract surgery is the most common operation in the UK. Exudative/wet age-related macular degeneration (AMD) and diabetic macular oedema (DMO) are two leading causes of sight loss in the UK, and these conditions may involve monthly eye injections into the vitreous cavity, “intravitreal injections.” With the increasing numbers of invasive eye procedures, the frequency of post-operative endophthalmitis (POE) though rare is increasing^1–5^.

The Royal National Institute of Blind People (RNIB) reported in 2009 that the financial endophthalmitis disease burden for cataract surgery would be £995,144,453^6^. The RNIB reported that although endophthalmitis is a very rare event, with increasing rates of cataract surgery, it is alarming that a significant percentage of patients will suffer serious loss of sight or loss of the eye, with an impact on quality of life, and also, the condition is often difficult to treat and can be very costly to manage ^6^.

The seminal study into the role of vitrectomy for POE following cataract surgery is the Endophthalmitis Vitrectomy Study (EVS) published in 1995^7^. The findings suggested that surgery to remove infected vitreous by pars plana vitrectomy (PPV) offered an improved outcome for individuals with vision limited to perception of light (POL) or poorer. The EVS found 33% of patients who underwent PPV with POL vision had visual acuity (VA) 20/40 or better at final follow-up, and 56% of patients achieved VA better than 20/100. The EVS assessed endophthalmitis treatment only in post-cataract surgery patients. No randomised controlled trial (RCT) evidence exists to answer the question of early vitrectomy surgery for the treatment of endophthalmitis following any eye surgery (retinal, glaucoma and corneal)^8^. In the last 10 years, vitrectomy surgery has evolved significantly with the introduction of small-gauge PPV (23-, 25- and 27-gauge), wide-angle viewing systems and the increased routine use of silicone oil, none of which were used in the EVS study. In the last 30 years, there have been a variety of POE management with vitrectomy surgery performed according to regional preferred practices based on clinical expert opinions, and these have fallen outside the EVS. In the real world, this timely implementation of an immediate vitrectomy surgery is variable based on the availability of theatre resources, so it can be immediate or delayed, with no standard approach in the absence of new robust RCT evidence^9–16^.

Endophthalmitis patients often spend far more than 6 months attending the hospital, requiring intensive and sustained medication. Early vitrectomy surgery has the potential to reduce the overall treatment period, reduce the time spent by patients attending the hospital for clinical and surgery appointments, reduce the risk of severe visual loss, and ultimately accelerate visual recovery. The psychological impact is also significant, with anxiety and depression a common feature, as well as considerable pain and vision loss. Societal costs could be reduced, including productivity losses for patients and their families ^6,17^.

The Moorfields ocular inflammation/medical retina services, which oversee the endophthalmitis hospital protocols, have already advocated earlier vitrectomy to be performed at vision levels better than the EVS standards. Given the increasing demand for earlier intervention for endophthalmitis, and advancements in vitreoretinal surgery, there are experts and clinicians globally raising awareness for a new trial to investigate the role of contemporary vitrectomy management of acute POE occurring as a complication of a larger spectrum of eye surgery or injection procedures ^8,15,16,18,19^.

The aim of early surgery to treat the whole spectrum of POE condition is to ultimately prevent severe visual loss across, and we hypothesised that performing early vitrectomy with intravitreal antibiotics (IVABs) compared to standard care, repeat IVABs in the management of POE may accelerate recovery and improve visual outcomes. With any new clinical trial, we would require to have engagement of affected patients, treating clinicians and the surgeons to address the question. The present study aims to assess the feasibility of conducting an RCT comparing early vitrectomy surgery to the current standard patient care. If we can confirm that such a trial is feasible, we would plan a definitive randomised controlled trial evaluating the effectiveness of early vitrectomy in the same patient population.

The EVIAN trial meets the primary endpoint, demonstrating feasibility in conducting a trial to randomise the target POE population to early vitrectomy surgery. Although not statistically significant, the effect on VA change indicates a potential meaningful benefit of vitrectomy with an acceptable safety profile compared to standard of care treatment.

## Methods

### Study design

We conducted an open-label, feasibility randomised, controlled study of *E*arly *V*itrectomy and *I*ntravitreal *A*ntibiotics for Post-operative Exogenous E*N*dophthalmitis (EVIAN study). Participants were randomised to a surgery group Arm A (early PPV surgery combined with a second IVAB injection, both performed within 48–96 h) and a standard of care Arm B (repeat IVAB treatments at 48 h. In Arm B, if the VA was a perception of light (POL) then a planned PPV could be performed at 12–15 days post-randomisation^20^. In the study design process, we conducted a Moorfields UCL Biomedical Research Centre patient and public involvement and engagement group forum, to gain feedback on our study objectives, target population, and study design. Our study group included two former POE patients (SC, CS) to ensure that all aspects of the research design study, trial conduct, patient care and management, and study oversight were optimised throughout the 4-year study.

### Study participants

The clinical trial was conducted across 21 National Health Service (NHS) ophthalmic centres in the United Kingdom. The trial was approved by the West Midlands-Solihull Research Ethics Committee (20/11/2020; 20/WM/0264), and conducted according to the Helsinki Declaration of 1975. Prior to enrolling participants, the study was registered at clinicaltrials.gov (NCT04522661, 13/08/2020). Participants were recruited from NHS centres that manage patients with POE with a specialist team of vitreoretinal surgeons, medical retina specialists, and uveitis specialists from the start of November 2021 to August 2024. Sixty-three participants with POE were recruited into the trial after providing their written informed consent. A Trial Steering Committee was appointed to independently monitor the progress of the trial and recommend prospectively whether there are any interim ethical or safety reasons why the trial should not continue. A Patient Advisory Group was appointed to address any ongoing patient issues, and support any future patient-related trial protocol amendments.

### Pre-study compliance

As part of standard NHS care, patients diagnosed with POE present at the eye clinic or emergency eye casualty. At the initial diagnosis of POE, the patient immediately underwent a standard of care vitreous biopsy (“tap”) and injection of IVABs. Potentially eligible POE participants were approached by the clinical team after their first presentation to the eye clinic, and an EVIAN study patient information sheet was provided to the patient by the doctor. They were given at least 24 h to reflect, consider their decision, with the opportunity to ask questions at that time or later. The patient was then instructed to attend at 48 h to be assessed, consented and then randomised into the EVIAN study. A numerical code was then assigned to each randomised participant.

### Inclusion and exclusion criteria

The inclusion criteria were: (1) over 18 years of age; (2) capacity to give informed consent; (3) not previously been enroled in this study in regards to their other eye; (4) confirmed diagnosis of POE at any time-point following an ocular surgery/procedure/injection; (5) symptomatic visual loss attributable to POE; (6) best corrected visual acuity (BCVA) worse than 35 ETDRS letters, including counting fingers, hand motions and POL vision; patient is healthy to undergo vitrectomy surgery. The exclusion criteria were: (1) major thromboembolic event within the past 3 months; (2) known adverse reaction to intravitreal antibiotics (amikacin/vancomyin/cephalosporins); (3) blood pressure greater than 200 systolic or 100 diastolic; (4) use a research investigational drug during the study; (6) optic atrophy in study eye; (5) Corneal oedema/haze that would prevent visualisation of fundus to perform vitrectomy surgery; any other condition that in the opinion of the investigator would preclude participation in the study (such as unstable medical status or severe disease that would make it difficult for the patient to be able to complete the study); patient over the age of 95 who present with POE should immediately be placed on the pre-screening log as ineligible.

### Randomisation procedure

At the 48-h visit, if the POE had either not improved, or the situation was deteriorating, with VA 35 ETDRS letters or worse, then the patient was eligible for the trial. The consent and randomisation occurred at this point. Once the patient completed enrolment, anonymous data was entered into the online Castor database. The electronic Sealed Envelope Company platform was used to randomise patients to groups at a 1:1 ratio to the Treatment (Arm A) or Control (Arm B). Block size was unknown to the investigators. This enrolment/randomisation visit was designated day 0 for the trial.

### Participant eligibility, screening and recruitment logs

The primary outcome measure for the EVIAN trial was the feasibility of conducting the specific type of trial within the designated population. We captured numerical and site-specific reasons/commentary data for ineligibility based on the total numbers of index POE cases each month (Supplementary Data 1 and 2). Sites provided reasons for participant(s) declining to participate and clinician engagement.

### Interventions

#### Arm A-vitrectomy surgery

Surgery was performed by a consultant vitreoretinal surgeon, or by a vitreoretinal fellow, within 48 h of randomisation. The choice of anaesthesia is dependent on the surgeon, patient choice and local standard practices. The PPV typically used a 3-port 27, 25, 23 or 20-gauge port technique. A second vitreous biopsy was taken before the infusion was started, and samples were sent to the site’s local microbiology and micropathology laboratory. A complete vitrectomy was performed; additional laser or cryotherapy treatment could be performed if there were retinal breaks present. The choice of intraocular tamponade agent was decided by the surgeon, and the choice of either air, gas or silicone oil all available options.

### Arm B-standard of care

An IVAB injection of two drugs: vancomycin (1.0 to 2.0 mg/0.1 ml) and, amikacin (0.4 mg/0.1 ml), or ceftazidime (2.25 mg/0.1 ml)) were administered on the day of randomisation. The injection was undertaken in the standard manner for the investigating unit, with topical anaesthesia. Participants with POE were treated according to the standard local hospital protocol for post-operative endophthalmitis. Each local site followed their respective endophthalmitis management protocol and this may result in different eye-drop treatment regimens, or different systemic/intraocular/periocular medications being used at different sites. This is satisfactory for this feasibility study.

In the control Arm-B, there was the option for a participant to undergo delayed vitrectomy surgery as part of the standard of care, within 12–15 days of randomisation. The criteria for intervention would be that the vision had reached POL, and a surgical discussion would be carried out in conjunction with the medical and surgical retina teams. Any patient who underwent vitrectomy surgery either for POE or a complication such as retinal detachment before this time point was excluded from the final analysis.

### Feasibility study objectives

#### Primary outcomes

Our study aimed to explore the feasibility and acceptability of carrying out an RCT of early surgical treatment compared to standard treatment for POE. The main future objective of this feasibility study was to determine whether a future definitive trial of early vitrectomy in POE would be feasible and use the EVIAN trial metrics to determine the sample size for a definitive trial. The major areas of uncertainty in this study relate to the acceptability of patients within an emergency setting to be randomised to either of two interventions, namely intravitreal antibiotics or early vitrectomy surgery. A further area of focus was the ability to identify, recruit and treat participants who have developed POE within the current NHS ophthalmic patient care pathway. In addition, we collected outcome measures at 6 months that include VA, adverse events/complications, and health economic data. Using VA outcomes, the study aimed to provide a signal of whether early PPV plus standard care IVAB injections showed better VA outcomes compared to standard care IVAB injections in the management of POE.

To determine the feasibility and effect size of a definitive RCT, the per-protocol primary outcome measures and success criteria included: (1) Assessment of patient recruitment, exclusion rates, retention rates, and drop-out rates; (2) Assessment of the willingness of clinicians to enrol patients into the trial; (3) To assess the willingness of participants to be randomised.

### Secondary outcomes

The following per-protocol additional secondary outcomes evaluated in the study included: distance BCVA; rate of completed follow-up; agreement of clinicians to enrol patients who will then be randomised to two arms; rate of secondary procedures; rate of complications; quality of life; and Client Services Receipt Inventory (CSRI) completion frequency and analysis. The following data would be used in a provisional exploratory analysis to identify factors (if any) predicting a beneficial outcome from early vitrectomy compared to the standard care treatment: Demographic data; type of eye surgery/procedure and/or intravitreal injection; baseline versus final VA; and type of culture-positive causative organism. All enroled study patients had an initial NHS standard treatment vitreous biopsy taken before randomisation, and this result was made available to the investigator at each site. During vitrectomy Arm A, a second vitreous biopsy was taken. The vitreous biopsy samples were analysed by local microbiology laboratories for culture and sensitivity analyses according to local microbiology protocols.

### Health economic measurements

The embedded health economics analysis assesses the feasibility of performing an economic evaluation of the intervention compared to the current standard treatment, using accepted methods^21^. We analysed the cost-effectiveness of early PPV surgery combined with a second IVAB injection, compared to standard of care treatment, using a short-run time horizon (6 months, the ‘within trial’ period). Costs were identified and assessed adopting the NHS and personal social care perspective. Costs included NHS resource use (e.g. antibiotics, treatments, diagnostic tests, surgery, further accident and emergency attendance, cost to treat complications and adverse events, etc), collected through the trial and using hospital data and CSRI^22^. Unit costs were taken from standard hospital coding sources. Patients in both groups might need additional care following the main treatment. The number and type of additional procedures was recorded and costed. The costs have been identified and checked with a clinical expert using the latest 2023/2025 NHS Payment Scheme (NHS England) ^23^.

The secondary health economics outcome measures in the trial were the visual function and the health-related quality of life (HRQL). These were measured by administration of the health-related National Eye Institute Visual Function questionnaire (NEI-VFQ-25)^24^ and the EuroQoL-5 Dimension questionnaires (EQ-5D-5L)^25^ to assess changes in quality-adjusted life years in both groups at 6 months. Patient-specific utility profiles were constructed assuming a straight line relation between each patient’s health-related quality of life scores at each follow-up point. The QALYs experienced from baseline to 6 months were calculated as the area underneath this profile. Cost-effectiveness was calculated as the mean cost difference between early vitrectomy and standard care divided by the mean difference in outcomes (NEI-VFQ-25/QALYs) to give the incremental cost-effectiveness ratio (ICER).

### Sample size

This feasibility study followed published recommendations that 24–70 participants are adequate to estimate a chosen parameter^26–28^. We used a 1:1 treatment-to-control ratio and targeted a total sample size of up to 64, allowing for some loss to follow-up. This would be sufficient to estimate the standard deviation of the outcome with up to 29 participants per group and to estimate the recruitment rate of 50% (expected 95% CI: 34–66%) if 128 eligible patients with acute endophthalmitis were approached.

### Statistical and reproducibility

Baseline characteristics were summarised as mean and standard deviation (SD) for approximately normally distributed continuous variables, median and interquartile range (IQR) for non-normal (skewed) variables, and count (%) for categorical variables; no baseline hypothesis tests were performed. Feasibility outcomes (recruitment rate and 24-week follow-up completeness) were reported with exact binomial 95% confidence intervals; no hypothesis testing was planned for feasibility outcomes. The exploratory between-arm comparison of change in ETDRS letters from baseline to 24 weeks used the Mann–Whitney *U*-test (two-sided, α = 0.05). We report medians (IQRs) by arm and associated *p* values. No hypothesis tests were prespecified in the protocol; this test was added post hoc to aid interpretation and is considered exploratory. Safety outcomes (adverse events and withdrawals) were tabulated as count (%) with exact 95% confidence intervals; no formal between-arm testing was planned. Participants were analysed in the groups to which they were randomised (intention-to-treat). Missing data were reported by the treatment group with reasons; no imputation was planned or performed. No subgroup analyses were prespecified. Any subgroup summaries of 24-week VA change were post hoc, exploratory, used the same non-parametric test within subgroups, were not adjusted for multiplicity, and are interpreted as hypothesis-generating. For VA measurement and analysis, the BCVA was recorded at participating sites in ETDRS letters at baseline and at 24 weeks. The analysis used these ETDRS values as provided; no Snellen or low-vision category (CF/HM/LP/NLP) data were used or converted. Distributions were assessed visually using histograms and Q–Q plots.

## Results

**Fig. 1:**
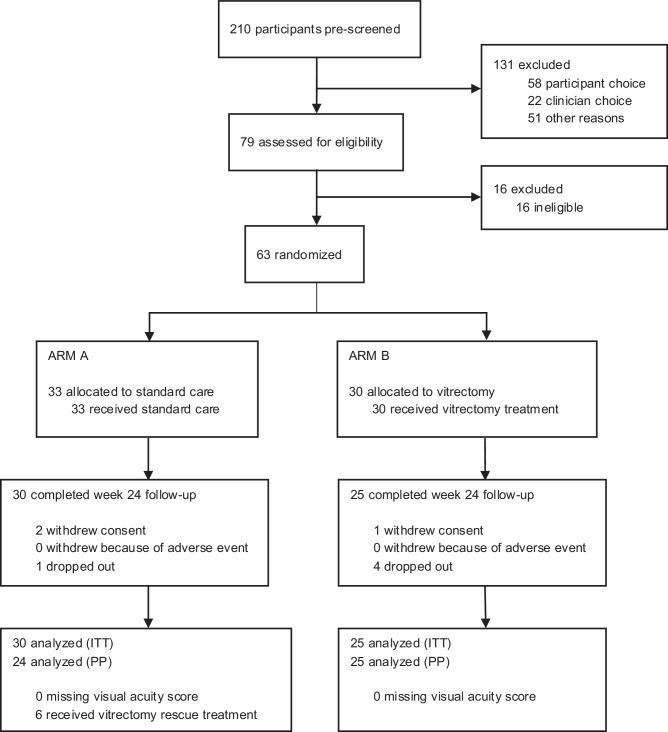
CONSORT diagram of trial design. A total of 210 participants were pre-screened for exogenous endophthalmitis meeting study criteria. We identified 70 eligible patients, and 63 were randomised into the trial. After exclusions, 55 participants completed the trial and are included in the final ITT analysis, and 49 are included in the PP analysis.

We identified a total of 210 patients with POE. We found 131 (62%) did not participate due to a number of factors, with 79 (38%) potentially eligible cases who agreed to participate. Over a 30-month recruitment period, we recruited 63 patients across 15 clinical sites, 30 in Arm A and 33 in Arm B, with an actual recruitment rate of ~79%. We excluded post-randomisation withdrawals of five participants in Arm-A (Vitrectomy) and 3 in Arm-B, resulting in 25 and 30 analysable data at 6 months for the planned ITT analyses. Another six patients who were initially allocated to Arm-B (control arm) but underwent an immediate/early vitrectomy surgery for complications of the underlying POE condition were excluded in the per-protocol (PP) analyses, resulting in 24 analysed at 6 months. See CONSORT diagram below for full details (Fig. 1).

The full list of patient characteristics for the whole study cohort is shown in Table 1. Participants had a similar distribution of demographic and ophthalmic characteristics across both treatment groups. Participants in the control Arm-B were more likely to be females (58 versus 50%), have phakic lens status (39 versus 30%), and have slightly more ocular co-morbidity (88 versus 83%), although there were similar proportions of patients affected by corneal disease, glaucoma, and AMD. In the total cohort (*n* = 63), the precipitating event of the POE was similar in both groups, in descending order of frequency, intravitreal injections, cataract surgery, vitrectomy surgery, glaucoma surgery, and one case after lens implant exchange surgery, with a mean duration of POE onset at 3 days in both Arms.

**Table 1:** Study patient characteristics

	Standard care (Arm B)	Vitrectomy (Arm A)	Overall
	(*N* = 33)	(*N* = 30)	(*N* = 63)
Demographic characteristics			
Age (mean, years)	72.8 (13.5)	70.7 (9.8)	71.8 (11.8)
Female	19 (58%)	15 (50%)	34 (54%)
Ethnicity			
White	28 (85%)	26 (87%)	54 (86%)
Asian	1 (3%)	4 (13%)	5 (8%)
Black	3 (9%)	-	3 (5%)
Other	1 (3%)	-	1 (2%)
Ophthalmic characteristics			
Study eye (left)	11 (33%)	14 (47%)	25 (40%)
Endophthalmitis cause			
Intravitreal injection	13 (39%)	14 (47%)	27 (43%)
Cataract surgery	9 (27%)	8 (27%)	17 (27%)
Vitrectomy surgery	4 (12%)	3 (10%)	7 (11%)
Glaucoma surgery	3 (9%)	1 (3%)	4 (6%)
Lens implant surgery	-	1 (3%)	1 (2%)
Other/unknown	4 (12%)	3 (10%)	7 (11%)
Duration of symptoms (days)*	3 [2–5]	3 [2,3]	3 [2–4]
Bacterial culture	23 (70%)	20 (67%)	43 (68%)
Lens status			
Phakic	13 (39%)	9 (30%)	22 (35%)
Anterior chamber lens implant	1 (3%)	1 (3%)	2 (3%)
Posterior chamber lens implant	19 (58%)	19 (63%)	38 (60%)
Other	-	1 (3%)	1 (2%)
Any ocular co-morbidity	27 (82%)	24 (80%)	51 (81%)
Corneal pathology	3 (10%)	2 (8%)	5 (9%)
Macular pathology	18 (62%)	14 (56%)	32 (59%)
Uveitis	2 (7%)	-	2 (4%)
Glaucoma	8 (28%)	5 (20%)	13 (24%)
Optic nerve disease	1 (3%)	-	1 (2%)
Other$	5 (17%)	10 (40%)	15 (28%)
ETDRS visual acuity (letter score)	3.1 (13.0)	1.1 (5.0)	2.1 (10.0)

Data were counted (%) unless indicated by an asterisk.

$Other ocular co-morbidity include the following: Standard care arm: amblyopia high myopia (*n* = 1), diabetic maculopathy/retinopathy (*n* = 1), epiretinal membrane (*n* = 1), wet age-related macular degeneration (AMD) (*n* = 1); Vitrectomy arm: diabetic retinopathy (*n* = 1), wet AMD (*n *= 1), scleral fixated lens implant extrusion (*n* = 1), herpes simplex (*n* = 1), hemi retinal vein occlusion (*n* = 1), central retinal vein occlusion (*n* = 1), retinal detachment (*n* = 1), unknown (*n* = 1).

*Skewed data summarised as median [IQR].

**Fig. 2:**
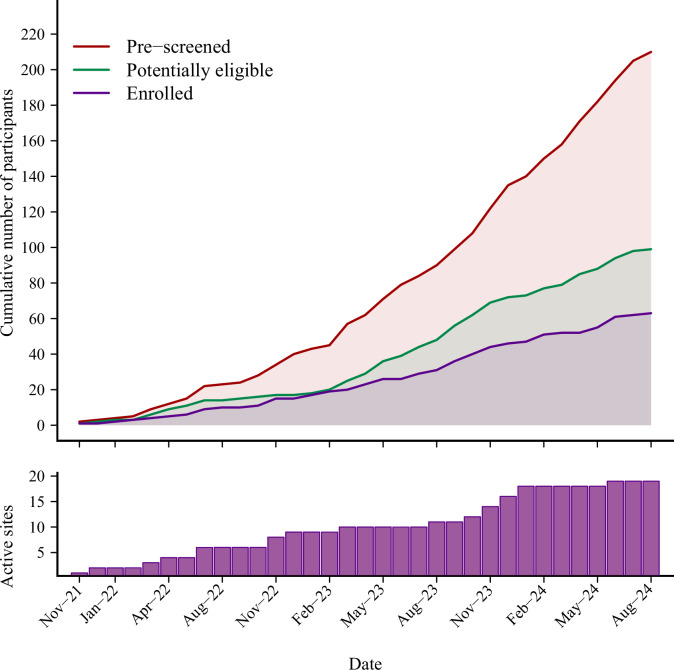
(Upper) Recruitment rates for the whole study population and final participant participation. (Lower) Clinical site activation over time. Over a 30-month recruitment period, we recruited 63 patients across 15/21 clinical sites, with 6/21 sites failing to recruit.

The key primary objectives and outcomes were achieved. The main reasons for non-participation (Supplementary Table 1) included participant choice (28%), clinician choice (10%), and participants deemed ineligible at the pre-screening phase (23%). “Other participant choice” reasons included does not wish to participate (31, 15%), anxiety of being in a clinical trial (3, 1%), and prefers standard of care (5, 2%). Clinicians decided the patient would benefit from immediate vitrectomy within 48 h (4, 2%), and the patient would be better off outside a clinical study (2, <1%). The remaining 63 participants were enrolled out of 70, with seven excluded upon full assessment for eligibility. Of the remaining recruited participants, 87% completed the trial, with the retention rate slightly lower in the vitrectomy group A (83%) compared to the standard care group B (91%). Nevertheless, only 73% of participants in the control arm B completed the trial per-protocol, after excluding those who received vitrectomy rescue treatment. We found that 21% participants made a choice not to enrol in the trial; however, a significant 79% of eligible participants agreed to participate, so we would deem that the EVIAN was feasible in achieving the primary objective. See Fig. 2 below.

**Fig. 3:**
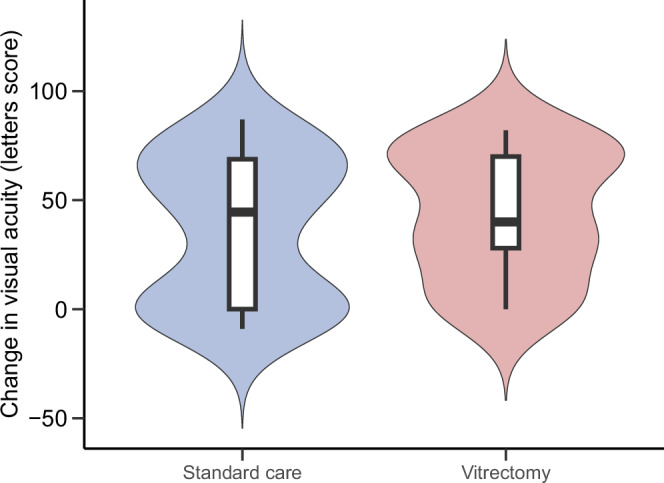
Violin plots show the kernel density of the distribution of each variable. Overlaid boxplots indicate the median (central line), interquartile range (box), and whiskers extending to 1.5 × IQR; observations beyond the whiskers are shown as outliers. Data were from the intention-to-treat population (standard arm, *n* = 30; vitrectomy arm, *n* = 25).

After the trial started in 2021, we had expected 21 clinical sites to be fully operational and recruiting patients, however post-setup, over the initial 12 months recruitment was below the expected rate. This was primarily due to slow site set-up that was triggered by post-COVID-19 pandemic restrictions on research capability/capacity at our designated clinical sites (November 2021 to January 2023). Once the site set-up rate increased, the recruitment rate increased according to our pre-study hypothesis. The expected recruitment rates then correlated with clinical site activation, as shown in Fig. 2.

The key secondary endpoints of the trial were visual function/VA changes, safety, and health economics feasibility evaluation.

**Fig. 4:**
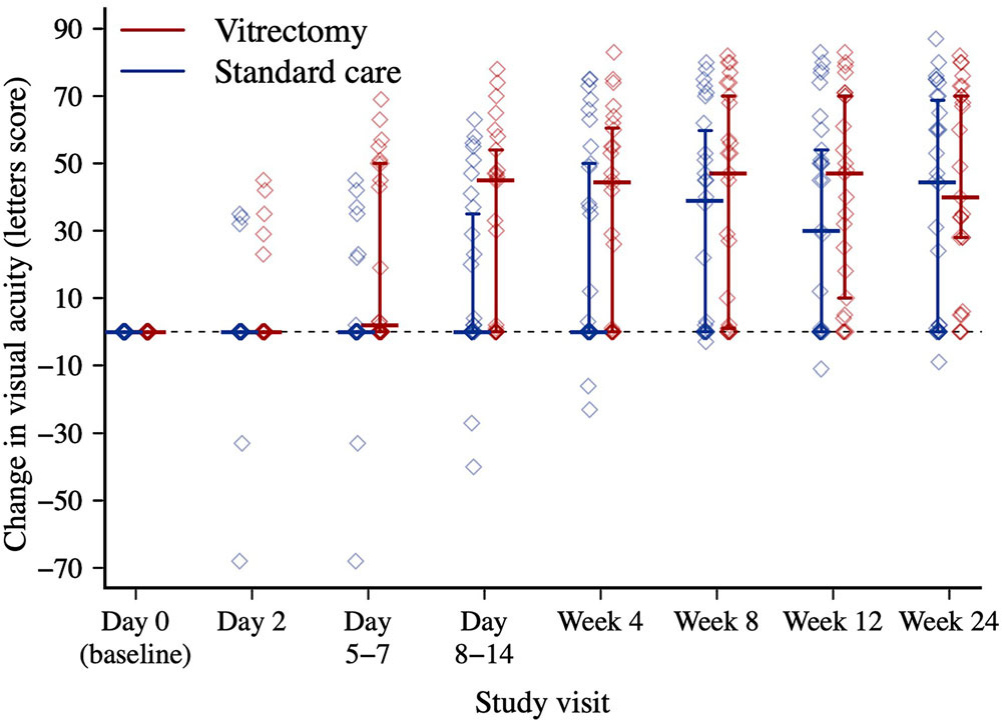
Point estimates represent the median change in visual acuity, with error bars indicating the interquartile range (IQR). Data were from the intention-to-treat population (standard arm, *n* = 30; vitrectomy arm, *n* = 25).

The ETDRS VA analysis at baseline for the whole group, Arm A (Vitrectomy, *n* = 30) and Arm B (Standard of Care, *n* = 33) was 1.1 ETDRS letters (5.0) for Arm A versus 3.1 ETDRS letters (13.0) for Arm B. This represents a two ETDRS letter difference. The overall VA at baseline for the whole group (*n* = 63) was 2.1 (10.0). There was no significant difference between the entry baseline VA, in the whole group at baseline, as this was randomised. We report the intention-to-treat (ITT) as the primary analysis for VA at 24 weeks and present the current per-protocol analysis as supportive, secondary analyses with justifications. As per-protocol, we aimed to explore the differences between standard of care treatment with delayed vitrectomy at day 12–15, versus early vitrectomy. For the whole group (Table 2), the mean change in VA from baseline to 24 weeks was 36.4 (SD 33.3) letters in Arm B (*n* = 30) versus 43.6 letters (SD 29.5) in Arm A (*n* = 25). The median change in VA (Fig. 4) from baseline to 24 weeks was 46.0 (IQR −0–70) letters in Arm B (*n* = 30) versus 40 letters (IQR 28–73) in Arm A (*n* = 25).

**Table 2:** Summary of trial primary and secondary outcomes

		Standard care (Arm B)	Vitrectomy (Arm A)	Overall
Primary outcomes				
Recruitment rate	(recruited/study period in months)	··	··	63/33 (1.91)
Exclusion rate	(excluded/assessed for eligibility)	··	··	16/79 (20%)
Retention rate	(completed trial/total recruited)	30/33 (91%)	25/30 (83%)	55/63 (87%)
Drop-out rate	(dropped out/total recruited)	3/33 (9%)	5/30 (17%)	8/63 (13%)
Complete PP*	(completed per-protocol/total recruited)	24/33 (73%)	25/30 (83%)	49/63 (78%)
Secondary outcomes (at week 24): Intention-to-treat				
ETDRS visual acuity (letter score)				
mean (SD)		39.8 (32.1)	45.0 (30.7)	42.1 (31.3)
median [IQR]		46 [0–70]	40 [28–73]	45 [5–70]
Change in ETDRS visual acuity (from baseline)				
mean (SD)		36.4 (33.3)	43.6 (29.5)	39.7 (31.5)
median [IQR]		44 [0–69]	40 [28–70]	44 [2–70]
Secondary outcomes (at week 24): Per-protocol				
ETDRS visual acuity (letter score)				
mean (SD)		35.8 (34.0)	45.0 (30.7)	40.5 (32.4)
median [IQR]		34 [0–70]	40 [28–73]	40 [2–70]
Change in ETDRS visual acuity (from baseline)				
mean (SD)		31.6 (34.9)	43.6 (29.5)	37.7 (32.5)
median [IQR]		13 [0–66]	40 [28–70]	35 [1–70]

*Per-protocol population include participants who completed week 24 follow-up and did not receive rescue vitrectomy therapy during the study period if initially allocated to the standard care arm (see Fig. 3).

**Fig. 5:**
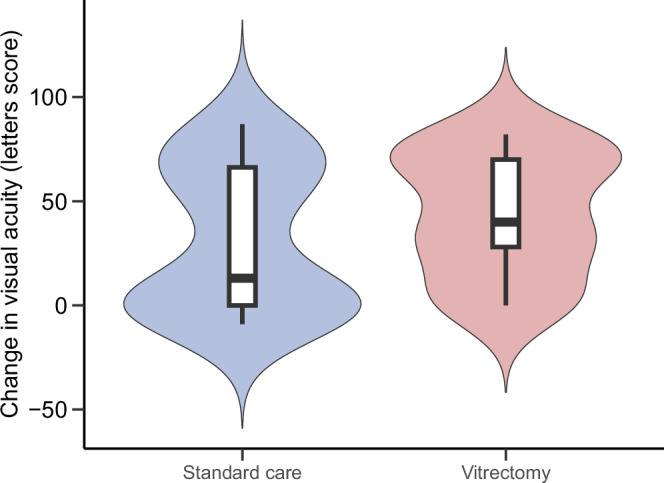
Violin plots show the kernel density of the distribution of each variable. Overlaid boxplots indicate the median (central line), interquartile range (box), and whiskers extending to 1.5 × IQR; observations beyond the whiskers are shown as outliers. Data are from the per-protocol population (standard arm, *n* = 24; vitrectomy arm, *n* = 25).

At 4 weeks, two patients in Arm B (Standard of Care) had immediate vitrectomy surgery within 7 days of randomisation that conflicts with the early vitrectomy intervention, and four participants had late vitrectomy after 4 weeks, which led to improved vision and was not part of the protocol design to test the effect of early vitrectomy. We report the per-protocol VA analysis of the final cohorts after exclusions (Arm-A, *n* = 25; Arm-B, *n* = 24). The baseline ETDRS VA was 4.2 letters (SD 15.2) in Arm A versus 1.4 letters in Arm B. There was no significant difference between the entry baseline VA. The VA at the final follow-up visit of week 24 does not follow a normal distribution (see Fig. 5 below).

**Fig. 6:**
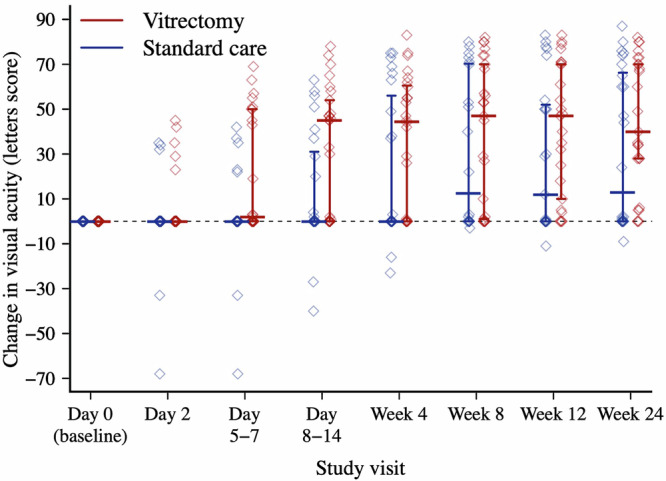
Point estimates represent the median change in visual acuity, with error bars indicating the interquartile range (IQR). Data were from the per-protocol population (standard arm, *n* = 24; vitrectomy arm, *n* = 25).

The median change in VA (IQR) per-protocol (Fig. 6) from baseline was 40 (28–70) letters in the vitrectomy Arm A group versus 13 (0–66) letters in the standard of care Arm B group. From baseline to week 24, we found a 27-letter improvement in VA scores in the vitrectomy Arm A compared to the standard of care treatment Arm B (*p* = 0.139). Although, the result was not statistically significant, the change in VA reveals a positive signal of effect, indicating a potentially greater effect on patient vision of early vitrectomy compared to standard of care treatment. See Fig. 6 below that shows the per-protocol change in median BCVA from baseline across time and treatment group. The sample size was too small to detect any significant differences between the two groups at 24 weeks.

As this is a feasibility study, the trial was not powered to detect significant differences in VA or other clinical outcomes. However, the results may provide valuable preliminary estimates of variability, which will be useful for informing sample size calculations for a future confirmatory trial.

### Adverse events/safety/additional procedures

We report the adverse events (AE) for the full cohort of 63 participants. The incidence of serious AE (SAE) were similar across treatment arms, 23 SAE in the vitrectomy Arm A group versus 12 SAE in the standard of care Arm B group. Arm B showed more participants with at least one non-serious AE (NSAE; 68 vs 47%). A key finding was the particularly higher incidence of retinal detachment in 8/33 cases (24.2%) in the standard of care Arm-B versus 2/30 cases in the early vitrectomy Arm-A (6.7%), (*p* = 0.118). These retinal detachment cases were associated with additional vitrectomy procedures being carried out during the trial that invalidated the randomised study intervention, hence those additionally treated patients were excluded. The NSAE tend to occur sooner than counterparts who received vitrectomy. See Table 3.

**Table 3:** Frequency of adverse events among treated participants

	NSAEs		SAEs	
	Standard care	Vitrectomy	Standard care	Vitrectomy
	(*N* = 33)	(*N* = 30)	(*N* = 33)	(*N* = 30)
Total number of events (n)	34	16	10	9
Number of events/participants				
Nil	14 (42%)	19 (63%)	25 (76%)	23 (77%)
1	10 (30%)	7 (23%)	6 (18%)	6 (20%)
2	6 (18%)	3 (10%)	2 (6%)	-
3+	3 (9%)	1 (3%)	-	1 (3%)
Days to first event*	14 [4–52]	35 [6–80]	42 [17–80]	34 [7–97]
Severity				
Mild	9 (27%)	6 (20%)	1 (3%)	-
Moderate	6 (18%)	4 (13%)	1 (3%)	3 (10%)
Severe	8 (24%)	2 (7%)	6 (18%)	4 (13%)
Relatedness to intervention				
Definite	1 (3%)	2 (7%)	-	2 (7%)
Probable	3 (9%)	3 (10%)	1 (3%)	-
Possible	3 (9%)	1 (3%)	-	-
Remote	5 (15%)	4 (13%)	-	1 (3%)
None	12 (36%)	3 (10%)	7 (21%)	4 (13%)
Expected event	12 (36%)	8 (27%)	5 (15%)	4 (13%)

Data were counted (%) unless indicated otherwise.

*N* indicates number of patients, *NSAE* non-serious adverse events, *SAE* serious adverse event.

*Skewed data were summarised as median [IQR]. Each participant is counted only once per event category, meaning the counts reflect the number of participants who experienced at least one event in that category (categories are not mutually exclusive).

**Fig. 7:**
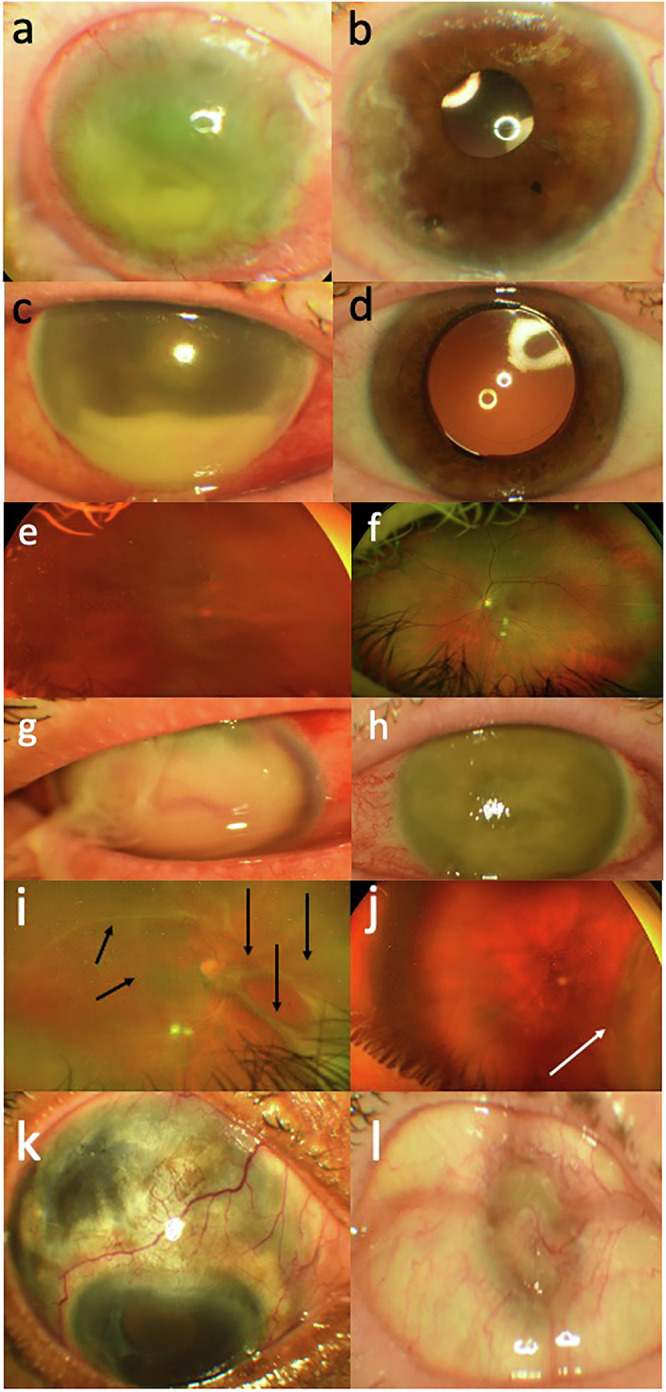
Anterior segment photograph examples of typical endophalmitis cases and sight-threatening complications,with widefield Optos photographic imaging. **a** Baseline endophthalmitis in Arm-B, **b** complete resolution at 6 months with standard of care treatment, **c** baseline endophthalmitis in Arm-A, **d** complete resolution at 6 months with early vitrectomy surgery, **e** widefield Optos fundus photograph of baseline endophthalmitis in Arm-A, **f** widefield Optos fundus photograph of completely resolved endophthalmitis with early vitrectomy surgery, **g**, **h** baseline endophthalmitis in Arm-A (**i**) Extensive macula and retinal tractional retinal detachments (black arrows) with ischaemia at 2 months following standard of care treatment, **j** rhegmatogenous retinal detachment (white arrow) with ischaemia at 4 weeks following standard of care treatment, **k** infectious necrotising scleritis at 6 months after standard of care treatment, in a patient with endophthalmitis after a mitomycin glaucoma tube operation, **l** phthisis bulbi at 6 months after standard of care treatment, in a patient with endophthalmitis after a vitrectomy and insertion of anterior chamber lens implant.

There were a higher number of complications following standard of care treatment, with severe sight-threatening complications reported that included phthisis bulbi, infectious necrotising scleritis, subretinal haemorrhage, hypotony, and iris rubeosis (Table 4). Retinal detachment was observed at almost x3.5 higher rate in standard of care treatment (24%) compared to the early vitrectomy group (6.7%). The phthisis bulbi case involved a Strept. pneumoniae organism, and Gram-negative bacilli was associated with cases with iris rubeosis, infectious necrotising scleritis, and cyclitic membrane. For the other serious complications, no organism was detected on ocular sampling culture. An overview of presentations and complications are shown in Fig. 7.

**Table 4:** Occurrence of ocular adverse events among treated participants

	Standard care (Arm B)	Vitrectomy (Arm A)
	(*N* = 33)	(*N* = 30)
Anterior segment		
Iris bombe	1 (3%)	
Anterior uveitis	3 (9%)	2 (6.7%)
Corneal oedema		
Iris rubeosis	1 (3%)	
Ocular hypertension	3 (9%)	2 (6.7%)
Cataract	2 (6%)	2 (6.7%)
Microbial keratitis	1 (3%)	1 (3.3%)
Glaucoma		1 (3.3%)
Infectious necrotising scleritis	1 (3%)	
Persistent Corneal epithelium defect	1 (3%)	
Posterior Segment		
Cystoid macular oedema	1 (3%)	1 (3.3%)
Retinal detachment	8 (24%)	2 (6.7%)*
Phthisis bulbi	1 (3%)	
Epiretinal membrane	1 (3%)	
Macular hole	1 (3%)	
Choroidal haemorrhage	1 (3%)	1 (3.3%)
Subretinal haemorrhage	1 (3%)	
Cyclitic membrane	1 (3%)	
Hypotony	2 (6%)	

*Analyses are based on the database locked on [31/01/2025]. One additional adverse event of retinal detachment was reported after lock, so three cases of retinal detachment (10%) in Arm A.

In the whole cohort, there was a relatively high vitreous biopsy positivity and yield rate in both groups (68%). Culture positivity and bacterial distribution were similar across groups in the final analyzed groups (Table 5), with a predominance of gram-positive cocci, particularly Staphylococcus epidermidis in the surgery group. On the other hand, Staphylococcus aureus, Streptococcus spp., and gram-negative bacteria are more common in the control group. In relation to the ocular complication of retinal detachment, we found six culture-positive cases. In the standard of care group (eight cases): *Streptococcus pneumoniae* (1); *Staphylococcus hominis* (1); *Staphylococcus aureus* (1); *Staphylococcus epidermidis* (1); *Haemophilus influenza* (1); with three cases undetected on culture. In the early vitrectomy group (2 cases), *Staphylococcus epidermidis* (1) and one case was undetected on culture.

**Table 5:** List of bacterial culture results

	Standard care	Vitrectomy	Overall
	(*N* = 23)	(*N* = 20)	(*N* = 43)
Culture-positive	19 (83%)	17 (85%)	36 (84%)
Gram-positive cocci (GPC)	16 (70%)	16 (80%)	32 (74%)
Coagulase-negative staphylococci (CoNS)	8 (35%)	14 (70%)	22 (51%)
Staphylococci (Staph). Epidermidis	5 (26%)	11 (61%)	16 (43%)
*S. capitis*	-	1 (6%)	1 (3%)
*S. warneri*	-	1 (6%)	1 (3%)
*S. haemolyticus*	1 (5%)	-	1 (3%)
*S. hominis*	2 (11%)	1 (6%)	3 (8%)
*S. aureus*	3 (16%)	-	3 (8%)
*Streptococcus* (Strept) spp.	5 (22%)	1 (5%)	6 (14%)
*S. pneumoniae*	2 (11%)	1 (6%)	3 (8%)
*S. parasanguinis*	1 (5%)	-	1 (3%)
*S. gordonii*	1 (5%)	-	1 (3%)
*S. oralis*	1 (5%)	-	1 (3%)
Other/unspecified GPC	-	1 (6%)	1 (3%)
Gram-negative bacteria (GNB)	3 (13%)	1 (5%)	4 (9%)
*Haemophilus influenzae*	1 (5%)	-	1 (3%)
Other GNB	2 (11%)	1 (6%)	3 (8%)

### Health economics outcomes

The cost of the surgery (Arm-A) interventions consisted of a pre-intervention IVAB, followed by vitrectomy surgery, biopsy and a second IVAB. The cost of the standard of care Arm-B consisted of a first pre-intervention VIAB, followed by a second VIAB. The cost of the standard treatment is £1078 per patient. On average, the additional treatment cost was around £384 per patient in the control group and £780 in the intervention group. A CSRI self-reported questionnaire has been administered at baseline and week 24 to assess the cost of healthcare resource use in the NHS.

On average, patients' costs for GP consultations are the same in the standard care Arm-B (£114.61) and the vitrectomy Arm-A group (£123.61). Pharmacy, hospital and community are also similar in both groups (Arm-B £104.94 vs Arm-A £112.93); the same for the optician (Arm-B £29.70 vs Arm-A £22.96) and day hospital (Arm-B £78.75 vs Arm-A £67.5). The main difference in cost was represented by A&E attendance, which is higher in the control group (Arm-B £311.98 vs Arm-A £183.17), and hospital inpatient visits which was higher in the surgery Arm-A intervention group (Arm-B £96.80 vs Arm-A £199.65).

Only medications relevant to the condition were included in the economic analysis (e.g. uveitis, endophthalmitis, corneal protection). On average, the cost of medication is higher in the surgery Arm-A group (£138.77) compared to the control Arm-B (£24.87). We do not think this is due to missing data, but probably the way patients have been instructed to report the medication taken or differences in patients’ severity.

Productivity losses were assessed using data on days of work lost captured through the CSRI and using the wages reported or a national ONS salary. In the vitrectomy intervention arm A, the majority of patients were retired, with only seven people currently working, and only six answered the questions about productivity loss. In the control Arm B, six people are currently working and filled the questionnaire. The productivity losses were higher in the control Arm-B compared to the intervention Arm-A (£263.22 versus £7.43). This reflects the age group of the POE patients undergoing cataract surgery, glaucoma surgery or intravitreal injections to manage AMD

At baseline, patients in the control Arm-B group have a higher utility mean score (0.70, SD 0.21) compared to those in the vitrectomy intervention Arm-A group (0.63, SD 0.29). While there is no difference in the mean utility in the two groups at 24 weeks (Arm-A 0.68, SD 0.28; Arm-B 0.68, SD 0.27), the overall QALY is lower for patients in the intervention arm (0.33 QALYs, SD 0.14) compared to standard of care Arm-B (0.35, SD 0.11). We only recoded the utility at baseline and 24 weeks, and importantly, we could not adjust the values for baseline. However, at 24 weeks, the utility in both groups is the same, meaning that patients in the early vitrectomy intervention Arm-A had an improvement in quality of life compared to those in the control arm-B. A full definitive phase 3 trial with a longer period of time of follow-up, and more endpoint-driven would provide more accurate estimates. Importantly, the EQ5D5L is a generic questionnaire and might not fully capture the quality of life in patients with vision problems.

Quality of life has been estimated using the NEI-VFQ-25, a self-reported questionnaire and consists of a base set of 25 vision-targeted questions representing 11 vision-related constructs, plus an additional single-item general health rating question. A higher score indicates better vision function and a higher quality of life. Patients in the early vitrectomy intervention Arm-A had a lower score at baseline (57.98, SD 22.44) compared to standard of care (62.72, SD 24.42). At 24 weeks, the score was higher in the early vitrectomy intervention Arm-A (67.85, SD 19.30) compared to patients in standard care (64.85, SD 23.21). However, on linear regression analysis, these differences between groups were not statistically significant at either baseline or 6 months.

## Discussion

The EVIAN trial reports that it was feasible to randomise patients who develop exogenous endophthalmitis following any eye procedure or eye surgery to undergo early vitrectomy at a vision level worse than 35 ETDRS letters. There were no unexpected safety concerns with early vitrectomy surgery in this patient cohort. In fact, adverse events, including sight-threatening retinal detachment, were lower after early vitrectomy surgery in this patient population. We report a positive signal of effect with visual acuity after early surgery intervention, with better visual outcomes.

The EVIAN trial was conducted to assess, to the best of our knowledge, for the first time, within an RCT, the willingness of patients with POE after any eye surgery or intervention to be randomised to early vitrectomy. Our study reports this is, in fact, feasible. We found that the clinicians are also willing to engage with the trial protocol, and treat patients with early vitrectomy according to the specified visual acuity thresholds. Given meeting the primary outcome of feasibility, without any significant safety signals from early vitrectomy surgery, we will be planning a definitive RCT to establish clinical efficacy of early vitrectomy in the same patient population, across a larger number of centres, according to the calculated sample size based on the EVIAN trial visual acuity outcomes.

There have been a number of studies exploring earlier surgery intervention, yet there are no studies investigating early vitrectomy across the full spectrum of POE^7–16^. The EVS study only included cataract surgery POE cases, and the surgery itself was either a core or limited vitrectomy that is no longer a standard approach by surgeons managing POE^7^. The FRIENDS study and recent Mexican trial showed early vitrectomy has positive effects on patient outcomes^5,16^. An Indian group reported an RCT for post-cataract surgery POE with lower vision thresholds, and immediate vitrectomy showed better VA outcomes compared to tap and inject^29^. However, POE with vision levels better than Snellen 60/ETDRS 35 letters is not usually treated by vitrectomy as part of the standard of care.

A number of centre-specific retrospective studies report better visual outcomes with early vitrectomy. The Manchester study found that PPV performed more than 24 h after presentation was associated with significantly better visual outcomes (OR 7.47)^30^. The European Vitreo-Retinal Society multicentric case series compared eyes treated with IVB alone versus early PPV within 1 week. The final VA did not differ significantly between groups^15^. A meta-analysis of studies published between 2010 and 2020 further showed the non-inferiority of IVB injection alone compared to PPV. The relative risk of gaining two or more lines of vision with PPV versus antibiotics alone was 1.04, suggesting comparable efficacy between the two strategies in postprocedural endophthalmitis, including post-cataract, post-injection and post-PPV cases ^31^.

The timing of vitrectomy surgery is important because the intravitreal environment associated with the natural history of endophthalmitis alters over time^32^. In the acute stages, there is mainly vitreous infective debris and opacification that responds well to vitrectomy surgery. In the subacute phase, starting from 7 days over several weeks, the endophthalmitis condition is associated with abnormal vitreoretinal adhesions and vitreoretinal traction can develop and worsen during this time. Our study reports that earlier vitrectomy surgery can lead to better visual outcomes. In our study, we observed a higher rate of retinal detachment in the standard of care arm, and this may reflect the progression of retinal ischaemia and thinning, with inflammatory fibrotic changes at the vitreoretinal interface, creating tractional retinal breaks, and subsequent retinal detachment. Early vitrectomy surgery will mechanically remove the vitreous bacterial load, clearing pro-inflammatory and toxic mediators, lowering retinal detachment risks. Early vitrectomy prevents the progression to a subacute stage, before the development of abnormal vitreoretinal adhesions and traction. We observed a lower incidence of ocular adverse events in the vitrectomy group for the whole spectrum of POE, and this again may reflect the mechanical clearance of the pro-inflammatory bacterial load at an early stage.

Our study produced a high yield of positive vitreous biopsy culture positivity (68%), similar to other studies^33,34^. The most common bacterial strain was gram-positive cocci in our study, again similar to other large studies by the French Institutional Endophthalmitis Study Group (FRIENDS) and EVS from the USA ^7,33,34^.

The visual outcomes were poorer in the standard of care treatment group, and we found a higher proportion of more virulent strains, such as Staph aureus and Streptococcus spp. in the control group^35–37^. Although none of these cases underwent a delayed vitrectomy for post-infective vitreous opacification, these organisms are recognised to carry a more negative prognostic factor. In fact, there was a higher rate of severe ocular complications in the standard of care group. In relation to the ocular complication of retinal detachment, in the standard of care group (eight cases): Streptococcus pneumoniae (1); *Staphylococcus* spp. (3); *Haemophilus influenza* (1); and in the vitrectomy group, *Staphylococcus* spp (1).

Our health economics evaluation had limitations. Patients in the surgery intervention Arm-A register a higher cost compared to patients in the control group, both from an NHS and societal perspective (£2,159 and £1968.50 more, respectively). This would be expected given the higher costs of day-case surgery. The quality of life of patients undergoing vitrectomy is lower at 24 weeks when measured through EQ5D5L (−0.02 QALYs in the intervention arm), but scores are better when using the NEI-VFQ-25 questionnaire (an extra three scores in the intervention arm). It is worth noting that the QALYs captured through the EQ5D5L might not be accurate in people with vision problems. The ICER, whilst using NEI-VFQ-25 to assess the quality of life, indicates an extra £719 to the NHS to improve the quality of life score obtained using the NEI-VFQ-25. As the NEI-VFQ-25 is more specific and relevant to this condition, we recommend using it in future larger trials, as it captures quality of life more accurately compared to the generic EQ5D5L. A micro-costing analysis of the cost of surgery was not performed for this study because the intervention was standardised and the tariff represented the intervention precisely. In a future full trial, we would run a micro-costing analysis of the protocol-driven surgery.

Regarding further key secondary exploratory objectives, we hypothesised that early vitrectomy surgery could potentially lead to a better VA outcome for patients compared to the standard of care.

The study reported VA analysis as a secondary exploratory outcome. We appreciate that any VA analyses may be biased for both ITT and per-protocol analysis. The sample size analyzed in the ITT was higher in Arm B compared to Arm A, so this is a confounding factor in the interpretation of the results. We reported the sample size for each analysis to be clear for any interpretation. Although the mean changes in VA from baseline to 24 weeks were more positive towards vitrectomy surgery (Arm A) for both ITT and per-protocol analysis, the VA at week 24 does not follow a normal distribution (see Figs. 3 and 6). We tested several data transformations, including log, square root, and inverse, and assessed their distributions visually. However, none of these transformations produced a normal distribution suitable for parametric testing. As a result, the Mann–Whitney *U*-test was applied to compare the median VA scores between the two treatment groups. Ultimately, this is a feasibility study and not powered for efficacy using the VA outcome measure. Any signals of effect cannot be drawn conclusively without a definitive trial. As per-protocol, we aimed to explore the differences between standard of care treatment versus early vitrectomy. We performed a post hoc median quantile regression (τ = 0.5) of 24-week change in ETDRS letters, adjusting for baseline VA (which is appropriate for non-normal outcomes). The between-group difference in median change remained similar, 24 letters in favour of early vitrectomy versus 27 letters unadjusted, so conclusions were unchanged. We highlight the per-protocol VA changes as these were, in fact, the most meaningful analyses to observe the true effects of early vitrectomy versus standard of care.

We report that the median improvement in VA from baseline to week 24, adjusted for baseline VA per-Arm, was 24 letters (five lines of ETDRS letters of VA) score in the vitrectomy Arm-A compared to standard of care treatment Arm-B. This improvement and difference in vision at 6 months would be deemed a clinically meaningful difference, and would be a positive signal of effect from a vitrectomy intervention for exogenous endophthalmitis. This signal of effect from the EVIAN trial differs from the EVS study, as the EVIAN patients were treated at lower levels of vision loss, Snellen worse than 6/60, rather than waiting for the patients to lose vision down to perception of light vision criteria. The lower vision entry criteria is reflected by clinicians who believe we should be carrying out surgical interventions at a lower VA threshold, thereby increasing patient access to surgery, so a greater number of patients with POE may benefit from surgery.

Our study reported the feasibility of randomising patients following any eye surgery/procedure across a wide geographical population, and the per-protocol outcomes following early vitrectomy showed a positive signal of effect for visual outcomes. Given the positive primary and secondary outcomes of the EVIAN trial, we believe that there is scope for planning a definitive multicentre, Phase 3 RCT to compare early vitrectomy surgery versus standard of care for POE after any type of eye surgery/intervention at VA worse than 35 ETDRS letters level.

## Supplementary information

Supplemental Information

Description of Additional Supplementary Files

Supplementary Data file 1

Supplementary Data File 2

## Data Availability

The individual participant data that underlie the results reported in this article, after deidentification (text, tables, figures and appendices), will be made available (including data dictionaries). Other documents that will be available include the study protocol, statistical analysis plan and informed consent form. The data will be made available 9 months and ending 36 months following article publication, to investigators whose proposed use of the data has been approved by an independent review committee (“learned intermediary”), with the purpose of individual participant data meta-analysis. Proposals should be directed to mahi.muqit1@nhs.net to gain access, and data requestors will need to sign a data access agreement with Moorfields Eye Hospital NHS Foundation Trust. Source data for Figs. 2–6 may be found in Supplementary Data Files 1 and 2.
