# Supplementary material for: Early vitrectomy and intravitreal antibiotics for post-operative exogenous endophthalmitis (EVIAN): a randomised control trial of feasibility
Source: Commun Med (Lond). 2026 Jul 16;6:419. doi: 10.1038/s43856-026-01664-w (PMC13421665; doi:10.1038/s43856-026-01664-w)
Supplement: Supplementary file 1 — Supplemental Information [file 43856_2026_1664_MOESM1_ESM.pdf]

	Approached (N=210)
Agreed to participate	79 (38%)
Did not participate	131 (62%)
Participant choice	58 (28%)
Does not wish to be randomised	4 (2%)
Anxiety of being in a clinical trial	3 (1%)
Prefers standard care process	5 (2%)
Does not wish to participate	31 (15%)
Does not wish to disclose	2 (<1%)
Other/multiple reasons	13 (6%)
Clinician choice	22 (10%)
Patient would benefit from immediate vitrectomy within 48hours	4 (2%)
Patient would be better off outside clinical study	2 (<1%)
Clinician opinion about lack of efficacy of treatment	-
Clinician feels that it is in the patient's best interest due to only eye status	-
Patient not informed by clinician	1 (<1%)
Other clinical reason	15 (7%)
Other	51 (24%)
Lost to follow up	3 (1%)
Patient is ineligible	48 (23%)

Supplementary Table 1:
Description of reasons for participation in study
