# Supplementary material for: Early vitrectomy and intravitreal antibiotics for post-operative exogenous endophthalmitis (EVIAN): a randomised control trial of feasibility
Source: Commun Med (Lond). 2026 Jul 16;6:419. doi: 10.1038/s43856-026-01664-w (PMC13421665; doi:10.1038/s43856-026-01664-w)
Supplement: Supplementary file 2 — Description of Additional Supplementary Files [file 43856_2026_1664_MOESM2_ESM.docx]

Description of Additional Supplementary Files

File name: Supplementary Data 1-2

Description: Source data for figures 2-6
